# An Event-Driven Classifier for Spiking Neural Networks Fed with Synthetic or Dynamic Vision Sensor Data

**DOI:** 10.3389/fnins.2017.00350

**Published:** 2017-06-28

**Authors:** Evangelos Stromatias, Miguel Soto, Teresa Serrano-Gotarredona, Bernabé Linares-Barranco

**Affiliations:** Instituto de Microelectrónica de Sevilla (CNM), Consejo Superior de Investigaciones Científicas (CSIC), Universidad de SevillaSevilla, Spain

**Keywords:** spiking neural networks, supervised learning, event driven processing, DVS sensors, convolutional neural networks, fully connected neural networks, neuromorphic

## Abstract

This paper introduces a novel methodology for training an event-driven classifier within a Spiking Neural Network (SNN) System capable of yielding good classification results when using both synthetic input data and real data captured from Dynamic Vision Sensor (DVS) chips. The proposed supervised method uses the spiking activity provided by an arbitrary topology of prior SNN layers to build histograms and train the classifier in the frame domain using the stochastic gradient descent algorithm. In addition, this approach can cope with leaky integrate-and-fire neuron models within the SNN, a desirable feature for real-world SNN applications, where neural activation must fade away after some time in the absence of inputs. Consequently, this way of building histograms captures the dynamics of spikes immediately before the classifier. We tested our method on the MNIST data set using different synthetic encodings and real DVS sensory data sets such as N-MNIST, MNIST-DVS, and Poker-DVS using the same network topology and feature maps. We demonstrate the effectiveness of our approach by achieving the highest classification accuracy reported on the N-MNIST (97.77%) and Poker-DVS (100%) real DVS data sets to date with a spiking convolutional network. Moreover, by using the proposed method we were able to retrain the output layer of a previously reported spiking neural network and increase its performance by 2%, suggesting that the proposed classifier can be used as the output layer in works where features are extracted using unsupervised spike-based learning methods. In addition, we also analyze SNN performance figures such as total event activity and network latencies, which are relevant for eventual hardware implementations. In summary, the paper aggregates unsupervised-trained SNNs with a supervised-trained SNN classifier, combining and applying them to heterogeneous sets of benchmarks, both synthetic and from real DVS chips.

## 1. Introduction

Deep learning and deep artificial neural networks (LeCun et al., 2015; Schmidhuber, 2015) currently hold the state-of-the-art performance in virtually any machine learning benchmark ranging from computer vision, speech recognition, natural language processing, audio recognition to name a few and in several cases they have surpassed human recognition rates (Schmidhuber, 2012; He et al., 2015). For these reasons they have been termed as one of the breakthrough technologies of our decade (MIT Technology Review, 2013), and have attracted a lot of attention from both academia and industry. Deep learning enables hierarchical feature extraction; with each additional layer the network learns to extract more abstract features, while it has been proven that by increasing the number of layers the classification performance improves (Hinton and Salakhutdinov, 2006). The vast number of operations these state-of-the-art deep neural networks require prohibits their execution on platforms with limited processing and energy resources. Currently, their execution is offloaded to remote computer clusters. However, this introduces additional communication overhead, which increases the overall system latency. For some applications, fast responses and low-energy consumption are crucial features as for example in mobile platforms, robotic, and critical systems.

Spiking Neural Networks (SNNs) (Gerstner and Kistler, 2002) have recently proven to be an interesting alternative to simulate large-scale neural networks (Izhikevich and Edelman, 2008; Ananthanarayanan et al., 2009; Eliasmith et al., 2012). SNNs are inherently asynchronous; similarly to biology, spiking neurons communicate through stereotypical events often referred to as spikes. Each spiking neuron updates its internal state upon receiving an incoming spike and generates an output whenever its membrane voltage crosses a threshold value. Recent advances in neuromorphic engineering (Mead, 1990) enable the emulation of SNNs directly on neuromorphic hardware in real-time (millisecond updates) or accelerated-time, with much higher efficiency in terms of power and speed compared to conventional computing platforms, despite the massive communication infrastructure overhead required. For example, the current state-of-the-art neuromorphic platform TrueNorth (Merolla et al., 2014) is capable of simulating a million spiking neurons in real-time while consuming 63 mW. The equivalent network executed on a high-performance computing platform was 100–200× slower than real-time and consumed 100,000 to 300,000× more energy per synaptic event^1^ (Merolla et al., 2014). TrueNorth is capable of delivering 46 billion synaptic operations per second per Watt (Sops/W) when executing at real-time and 70 billion Sops/W at 5× faster than real-time (200 μs updates). An additional advantage of simulating SNN on neuromorphic platforms is that their event-based nature makes them more suitable to use with low-power, low-latency, high dynamic range neuromorphic vision and auditory sensors (Lichtsteiner et al., 2008; Liu et al., 2010; Lenero-Bardallo et al., 2011; Posch et al., 2011, 2014; Serrano-Gotarredona and Linares-Barranco, 2013).

SNNs have been characterized as the 3rd generation of artificial neural networks (ANNs) and while they are theoretically computationally more powerful than conventional continuous or rate-based ANN (Maass and Markram, 2004) they still lack the success of their predecessors. A possible explanation for this is the lack of sophisticated training algorithms like those that have been developed for ANNs over the past decades. Because the activation functions of spiking neurons are not differentiable (due to the threshold condition), SNNs are not able to directly use the popular training methods used in ANNs, such as backpropagation, which require differentiable functions. To address this, research groups currently focus on two different paths: either employ biologically plausible unsupervised learning rules, like spike-timing-dependent plasticity (STDP) (Dan and Poo, 1992) to extract features from inputs (Masquelier and Thorpe, 2007; Bichler et al., 2012; Neftci et al., 2014; Diehl and Cook, 2015; Kheradpisheh et al., 2016) or they follow an intermediate step: a neural network is trained off-line using continuous/rate-based neuron models (ANNs) with state-of-the-art supervised training algorithms (LeCun et al., 1998; Hinton et al., 2006) and then map the trained network to a SNN (Merolla et al., 2010; O'Connor et al., 2013; Pérez-Carrasco et al., 2013; Diehl et al., 2015), ready to be executed efficiently on a neuromorphic platform (Camuñas-Mesa et al., 2010; Arthur et al., 2012; Furber et al., 2014; Merolla et al., 2014; Stromatias et al., 2015a).

While the current state-of-the-art results in classification tasks with SNNs come from the latter methodology (Diehl et al., 2015; Rueckauer et al., 2016) there are a number of drawbacks that are usually not addressed. One major issue is that these neural networks are trained using synthetic data. This is, the input spiking activity fed to the SNN is generated artificially from frame images (like MNIST), where the gray level of an image pixel is mathematically transformed into a stream of spikes using some algorithmic method (like the popular Poisson distribution encoding). This imposes practical issues when switching to non-synthetic real input data captured with a physical spiking silicon retina such as a neuromorphic dynamic vision sensor (DVS) (Lichtsteiner et al., 2008; Posch et al., 2011; Serrano-Gotarredona and Linares-Barranco, 2013). In this case, the time distribution of spikes/events coming from these sensors are not Poissonian and this results in the SNN performing very poorly in terms of classification accuracy. Our personal experience is that when mapping the full network from ANNs to SNNs the loss in accuracy is low/reasonable if input data is generated synthetically as Poisson spike distributions. However, accuracy drops dramatically if input data to the same network is replaced by real sensory data recorded from spiking silicon retinas (like N-MNIST, MNIST-DVS, Poker-DVS) (Orchard et al., 2015a; Serrano-Gotarredona and Linares-Barranco, 2015; Soto, 2017). As a matter of fact, the reported high accuracy works on training a full network in the frame-domain and mapping it to the SNN domain always report results with synthetic data (Merolla et al., 2010; O'Connor et al., 2013; Diehl et al., 2015)

On the other hand, work presented in recent years has shown that it is possible to learn features efficiently with SNNs using STDP (Masquelier and Thorpe, 2007; Bichler et al., 2012; Diehl and Cook, 2015; Kheradpisheh et al., 2016) and other unsupervised methods such as event-based Contrastive Divergence (CD) (Neftci et al., 2014, 2016). An advantage of leaning with STDP is that it inherently takes into consideration the timing distribution of events coming from a DVS sensor (Bichler et al., 2012; Roclin et al., 2013). However, after extracting the SNN features many researchers use a method to convert the asynchronous events of an SNN to frames for an ANN in order to train a frame-based classifier such as a Support Vector Machine (SVM) (Kheradpisheh et al., 2016) or Radial Basis Function (RBF) classifier (Masquelier and Thorpe, 2007) and evaluate how “good” the spike-domain learned features are. Often this conversion is done by a population of leaky integrate-and-fire (LIF) neurons with infinite threshold (Masquelier and Thorpe, 2007). The neurons integrate the inputs from the previous layers and when a control signal arrives they export their internal states and a frame is created. In a practical SNN system, for example if fully deploying it as a compact hardware, it is highly desirable that all stages, including the classifier, can be implemented in the spiking domain.

Recent work has successfully explored direct training in the spiking domain. For example, Lee et al. (2016) proposed a backpropagation-like technique for directly training a multi-layer SNN with fully-connected inter-layer connectivity. They used real DVS recorded (N-MNIST) input sensory data (Orchard et al., 2015a) and report the best accuracy reported to date with a FC (fully-connected) topology and DVS data (98.66%). Neftci et al. (2017) have recently proposed a simple event-driven random backpropagation rule to rapidly learn deep representations, although they only provide results for synthetic input data.

In this paper we present an alternative methodology for training only an event-based classifier in a supervised manner. This spike-based classifier can be used as the output layer in any SNN that has already extracted features, for example using STDP or another unsupervised method (Bichler et al., 2012; Roclin et al., 2013; Neftci et al., 2014, 2016; Diehl and Cook, 2015; Kheradpisheh et al., 2016), or to fine-tune an already trained SNN (O'Connor et al., 2013). The method proposed here is based on the idea of training in the frame domain and then testing with events but instead of training a full ANN and then mapping it to an SNN, it uses the spiking output activity of the (pre-classifier) SNN to create a new frame-based dataset, which captures the dynamics of the spikes. These SNN-sensitive frames are then used to train a fully-connected classifier, using supervised learning algorithms such as stochastic gradient descent (SGD) (Bottou, 2010) on the new dataset. After training, the frame-based ANN classifier is mapped directly to a population of LIF neurons, which is used as the output layer of the SNN. The advantages of this method is that it is easy to implement by taking advantage of popular supervised training algorithms, it yields good prediction accuracy for both synthetic data and real DVS data, and it can cope with neuron leakages with minimal loss in the classification performance. To our knowledge, this technique of training the classifier output layer of an SNN has never been reported before^2^.

This paper is structured as follows: Section 2 discusses the datasets used for this work, which include both data recorded from a DVS sensor and synthetically generated spike-trains from static images. Section 2.2 introduces the SNN simulator used for this work. Section 2.3 describes the topology of the neural network used for all the experiments. Section 2.4 presents the neuron and synapse model. Section 2.5 describes the proposed method to train a classifier in a supervised manner using frames and how to convert it back to an SNN. Section 3 presents results and finally, Section 4 presents some discussions and the conclusions.

## 2. Materials and methods

### 2.1. Data sets

For this work we used two types of data sets with different encoding methods: MNIST handwritten digit data sets and poker card deck data sets recorded with DVS cameras.

The original MNIST data set (LeCun et al., 1998) consists of 70,000 28 × 28 gray scale images of handwritten digits out of which 60,000 digits are used for training and 10,000 for testing. In this paper we use 4 variants of the MNIST data set. The first two convert the original MNIST static images into artificial spike-trains using each a different method (Liu et al., 2016), (a) Poisson encoding and (b) intensity-to-latency encoding. The other two utilize spikes recorded from a DVS sensor and are known as (c) MNIST-DVS (Serrano-Gotarredona and Linares-Barranco, 2015) and (d) N-MNIST (Neuromorphic MNIST) (Orchard et al., 2015a).

**Figure 1:**
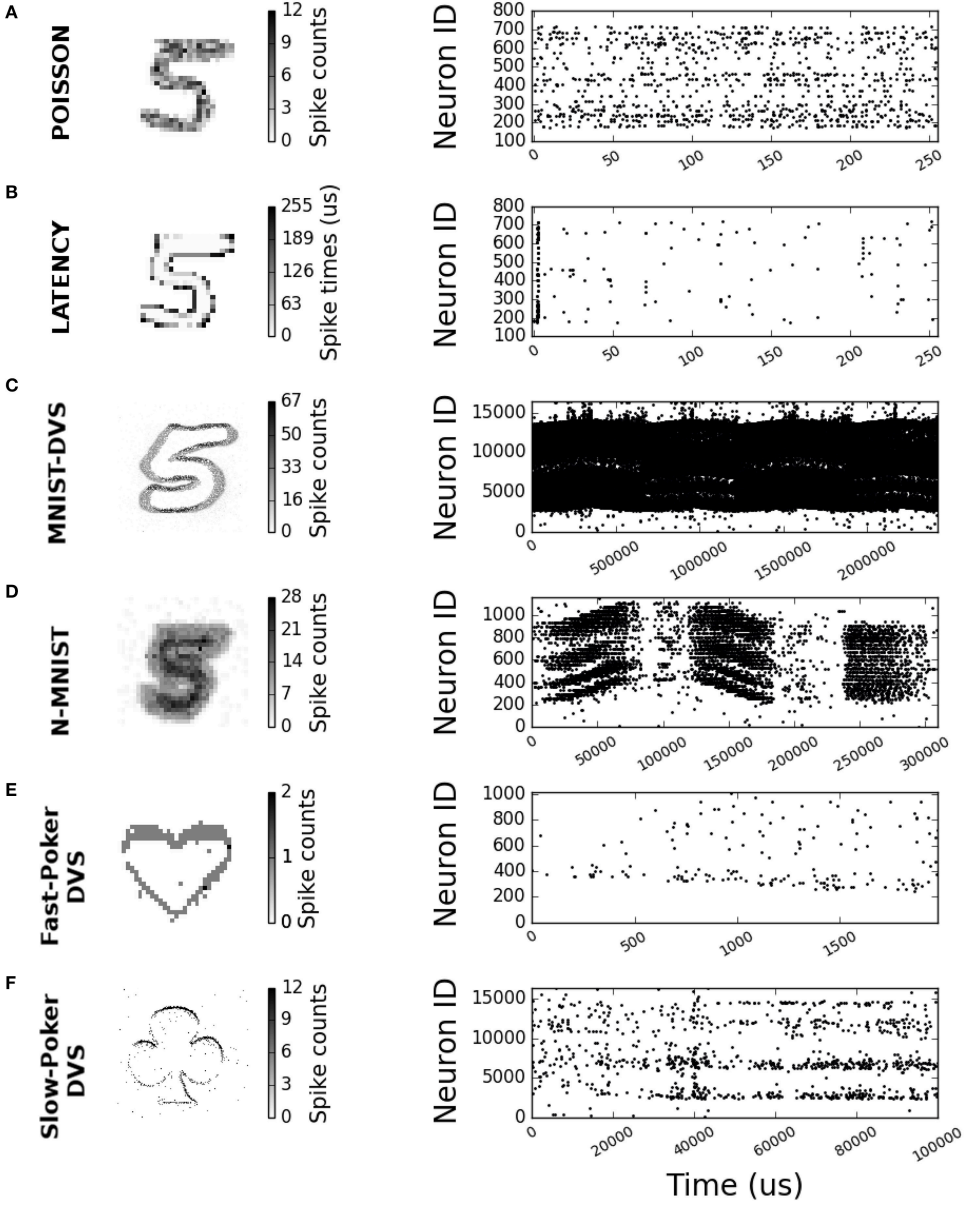
2-D histograms and raster plots for different encoding schemes and neuromorphic data sets. **(A)** Poisson 28 × 28 input size sample. **(B)** Latency 28 × 28 input size sample. **(C)** MNIST-DVS 128 × 128 input size sample. **(D)** N-MNIST 34 × 34 input size sample. **(E)** Fast-Poker DVS 32 × 32 input size sample. **(F)** Slow-Poker DVS 128 × 128 input size sample.

The poker card data sets consist of either (e) browsing at very high speed a poker card deck in front of a DVS sensor (Serrano-Gotarredona and Linares-Barranco, 2015), or (f) showing printed symbols on paper to a DVS (Soto, 2017). Next we briefly describe the different data sets:

- **MNIST Poisson Encoding**. This is the most popular method of converting static images into spike-trains and has been used in several published works (O'Connor et al., 2013; Diehl and Cook, 2015; Diehl et al., 2015; Rueckauer et al., 2016; Stromatias et al., 2015b). Each pixel of an MNIST digit is converted to a Poisson spike train with a firing rate proportional to its intensity, while all pixels' firing rates are scaled such that the total number of spikes of the input population is fixed for a given stimulus duration. This is illustrated in Figure 1A, where maximum spikes per pixel was set to 15, total spikes to 1,000, and stimulus duration to 255 μ*s*.
- **MNIST Intensity-to-Latency Encoding**. In this method only one spike per pixel is generated, based on its intensity (Masquelier and Thorpe, 2007; Kheradpisheh et al., 2016). Pixel intensity is transformed linearly into an event delay with respect to a common reference instant (time “0” in Figure 1). Pixels with higher intensity will produce an earlier spike. The advantage of this method is that, since it produces only one spike per pixel, it will theoretically result in lower activity and faster response times than Poisson encoding. Figure 1B shows an example of intensity-to-latency encoding, where maximum intensity is encoded with 0 delay spikes and zero intensity is encoded with 255 μ*s* delay spikes.
- **MNIST-DVS data set**. The MNIST-DVS data set is a version of the original MNIST data set recorded with a DVS sensor (Serrano-Gotarredona and Linares-Barranco, 2015). This data set consists of a 128 × 128 input size set of 30,000 DVS camera recordings. Each recording is obtained by displaying a slowly moving symbol from the standard MNIST database on an LCD monitor for about 2–3 s. Due to the size of the recorded files, only 10,000 of the original 70,000 symbols were recorded, but each symbol was displayed at three different scales. For this work the 10,000 samples recorded at higher scale (called “Scale 16” in Serrano-Gotarredona and Linares-Barranco, 2015) were used. From these, 8,000 samples were used for training and the remaining 2,000 samples were used for testing. The MNIST-DVS data set includes some Matlab scripts for optionally pre-processing the recorded events. This pre-processing can eliminate the 75 Hz LCD screen refresh rate harmonic and/or stabilize the moving digit. For this work we did not stabilize the digits, as we were interested in moving samples. Also, we noticed that removing or not the 75 Hz LCD screen refresh rate was absolutely irrelevant for recognition performance (the results were identical, whether or not this harmonic was removed).
- **N-MNIST data set**. The N-MNIST data set imitates biological saccades for recording the complete MNIST data set with a DVS sensor. A DVS sensor is mounted on a pan-tilt unit pointing to a monitor where digits are displayed. The DVS is then subject to 3 sequential 100 ms saccades at 3 different angles (horizontal, +60°, and −60°). Therefore, each sample corresponds to 3 saccades with a total duration of about 300 ms.
- **Fast-Poker-DVS data set**. The Fast-Poker-DVS data set was created by browsing poker card decks in front of a DVS camera (Serrano-Gotarredona and Linares-Barranco, 2015). Each card crossed the field of view in about 10–30 ms. The poker pips were tracked and isolated to a 32 × 32 pixel window. The data set contains a total of 131 symbols. Another variant of this data set with 40 cards has also been used for comparison with previous works (Pérez-Carrasco et al., 2013).
- **Slow-Poker-DVS data set**. In this data set (Soto, 2017) recordings were made while a human was holding a poker symbol in front of a DVS moving it at “human speed” (Soto, 2017). The purpose of this data set was for setting up interactive demos. This data set consists of four different DVS sensor recordings of around 3 min duration each. Each recording corresponds to a poker card symbol (club, diamond, heart or spade). To create a training and testing data set, the recordings were split into 100 ms time slots. This resulted into a total of 6,751 samples, 5,402 (80%) of which were used for training and 1,349 (20%) for testing.

### 2.2. Spiking neural network simulator

For this work we used the *Modular Event-Driven Growing Asynchronous Simulator* (MegaSim)^3^ as our main simulation platform. MegaSim is a tool designed for simulating Address-Event-Representation (AER) (Mahowald, 1994) multi-module hardware systems behaviorally, with strong emphasis on hardware performance parameters of the modules (processing delays, handshaking and communication delays, parameter variations, noise, etc.).

In MegaSim the user defines a netlist of *modules* interconnected through AER links (also called “*nodes*”). Modules process events coming in on their input AER nodes and generate output events on their output AER nodes. The netlist also contains at least one “*source node*,” which provides a list of time-stamped events (from a DVS recording, or created artificially). Modules in MegaSim can have an arbitrary number of input/output ports and can either be populations of neurons, an algorithm described in standard C programming language or combinations of both. At start-up only the events in the source nodes are available, which are tagged as “*unprocessed*.” MegaSim looks at all nodes, picks the earliest un-processed event, tags it as “*processed*,” calls the modules that receive it, and performs the corresponding processing within each module. If a module generates events at one or more of its output ports, they are written on those nodes with a timestamp equal to the actual time or equal to some future time in case the module is modeled as having some processing delay, while tagging them as “*unprocessed*.” Every time an event is added to the list of unprocessed events of a node, all the unprocessed events are re-sorted according to their timestamps.

AER events are represented using 3 timing values and *n* event parameters. Event parameters are typically 3 and are in the form of X, Y, and polarity. However, these parameters can be any signed integer and the user decides how a module interprets and processes them. The 3 timing parameters include: pre-request (*pre-Rqst*) which represents the time an event is created inside a module, request (*Rqst*) which is the time when this event is actually put on the node, and acknowledge (*Ack*) which is the time when the acknowledgment signal is communicated. When an event has been queued in a link or node, it is tagged as “*unprocessed*” by setting *Rqst* and *Ack* to -1. Once *Rqst* and *Ack* have both a positive number, it means they are tagged as “*processed*.”

The simulation finishes by either setting a maximum simulation time, or when there are no more “*unprocessed*” events. When the simulation finishes there will be a list of timestamped processed events for each node in the network. Activity of nodes can then be visualized with a dynamic event viewer like jAER (Delbruck, 2013).

### 2.3. Network architecture

#### 2.3.1. Convolutional neural network

The goal of this work focuses on training and using an efficient event-driven classifier. A classifier requires a previous feature extraction sub-system. For this work we used a Spiking Convolutional Neural Network to extract features before training the classifier. Convolutional Neural Networks (ConvNets) (LeCun et al., 1998) are multi-layer feed-forward neural networks that are composed of alternating layers of convolution and spatial sub-sampling, with non-linearities between subsequent layers. Each convolutional layer is structured into a number of “Feature Maps,” each detecting a specific feature. ConvNets introduce three basic ideas: local receptive fields, shared weights, and pooling.

Neurons in a convolutional layer are connected only to a sub-region of the layer before it (local receptive field), instead of to all presynaptic neurons as in a fully-connected network. Inside a Feature Map, the connectivity pattern and synaptic weights of the local receptive field is the same for all neurons. Consequently, synaptic weights are shared by all neurons in the same Feature Map, and all neurons will therefore detect the same features but at different locations within the Feature Map. Some of the advantages of the shared weights per Feature Map is that they greatly reduce the number of learning parameters, which also reduces the memory requirements, and also results in speed-ups during the training process when compared to fully-connected neural networks (LeCun et al., 1998).

A pooling layer is added periodically in-between successive convolutional layers. Pooling acts as a non-linear down-sampling that reduces the spatial size of the representation, the computations for the upper layers and finally provides a form of translation invariance. For this work we used subampling as a pooling layer.

**Figure 2:**
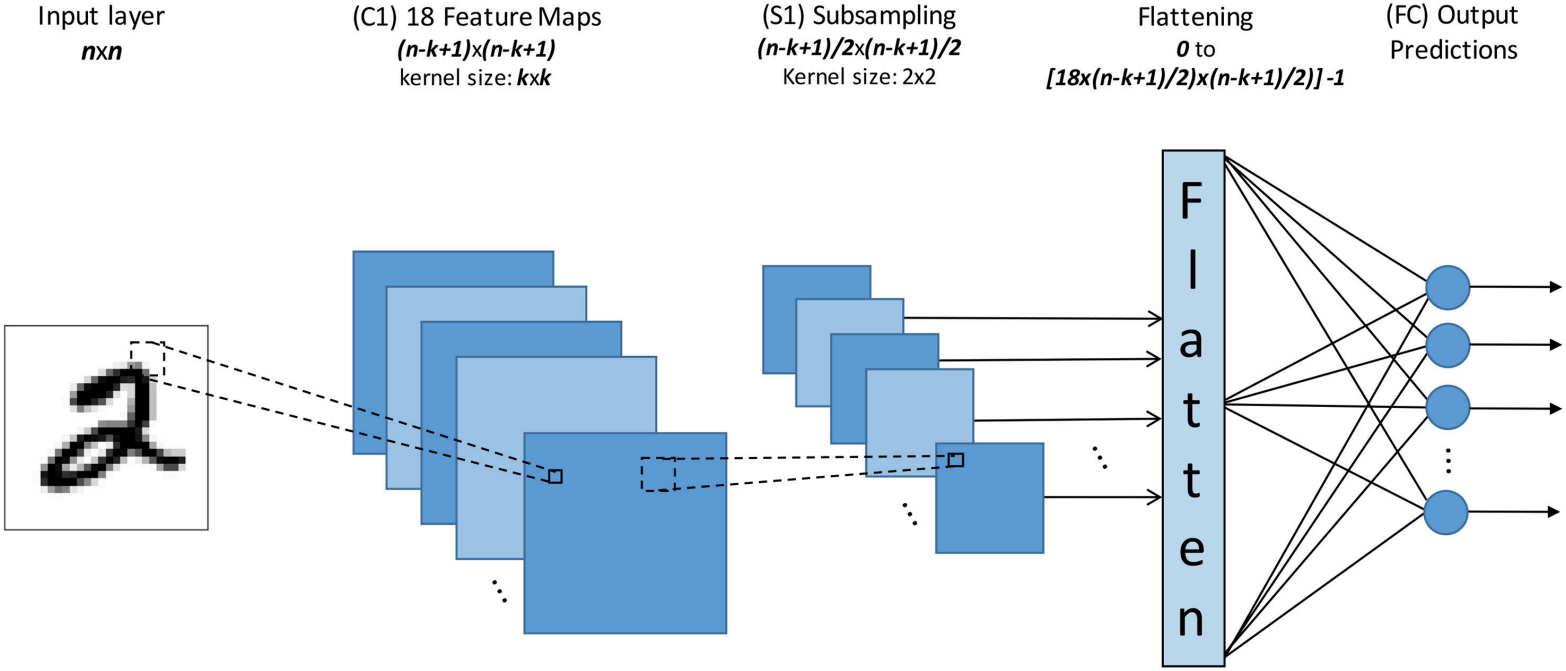
The topology of the event-driven Convnet used for this work. Where *n* is the size of the input layer, *k* is the size of the kernel.

Figure 2 shows the Spiking Neural Network (SNN) topology we have used for all our experiments. The input field size (*n*×*n*) varies depending on the data set from 28 × 28 to 128 × 128. Inputs are fed to a one-layer ConvNet, consisting of 18 Feature Maps (C1) of size (*n* − *k* + 1) × (*n* − *k* + 1) each with a receptive field of size *k*×*k* (also called “convolutional kernel”), followed by a subsampling pooling layer (S1).

The output of this 1-layer ConvNet consists therefore of 18×((*n* − *k* + 1)/2)×((*n* − *k* + 1)/2) neurons, which are rearranged into a one-dimensional vector by a module named “Flatten.” The outputs of this Flatten module provide the inputs to our fully connected spiking classifier. For example, for an input image of 28 × 28 and a convolutional kernel size of 7 × 7 the output of this 1-layer ConvNet consists of 2,178 neurons. The 2,178 outputs of this module are fully connected to all the classifier output neurons (one for each class).

**Figure 3:**
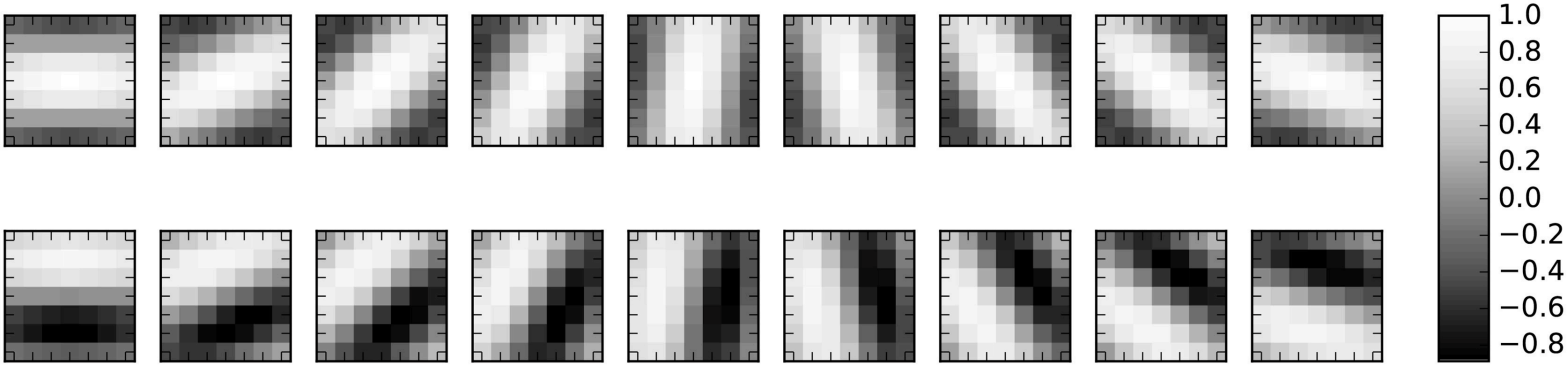
The pre-computed 7 × 7 gabor kernels used for the first convolutional layer.

The 1-layer feature extraction ConvNet was always programmed with 18 Gabor Filter kernels. An example of the Gabor Filter kernels with size 7 × 7 is shown in Figure 3. These kernels were generated using the following equations

(1) $$\begin{array}{l} g(x,y,\lambda ,\theta ,\psi ,\sigma ,\gamma )=\exp(\frac{-x^{\prime 2}+\gamma^{2}+y^{\prime 2}}{\sigma_{y}^{2}})\times \\ \;\;\;\;\;\;\;\;\;\;\;\;\;\;\;\;\;\;\;\;\;\;\;\;\;\;\;\;\;\;\;\;\;\;\cos(2\phi \frac{x^{\prime }}{\lambda }+\psi ) \end{array}$$

(2) $$\begin{array}{l} x^{\prime }=x\cos\theta +ysin\theta ,\\ y^{\prime }=-x\sin\theta +ycos\theta^{\prime } \end{array}$$

where λ is the wavelength of the sinusoidal factor, θ is the orientation of the normal to the parallel stripes of a Gabor function in degrees, ψ is the phase offset, σ represents the width of the Gaussian, and γ is the spatial aspect ratio. For the 18 kernels we used 9 orientations and 2 phases. The parameters used to generate the 2D Gabor kernels are shown in Table 1.

**Table 1:** The parameters used to generate the 18 2D Gabor kernels.

**Parameters**	**Values**
θ	[ 0, 20, 40, 60, 80, 100, 120, 140, 160]°
σ	4
λ	8
ψ	[0.0, 1.7]
γ	0.5

#### 2.3.2. Fully connected network

**Figure 4:**
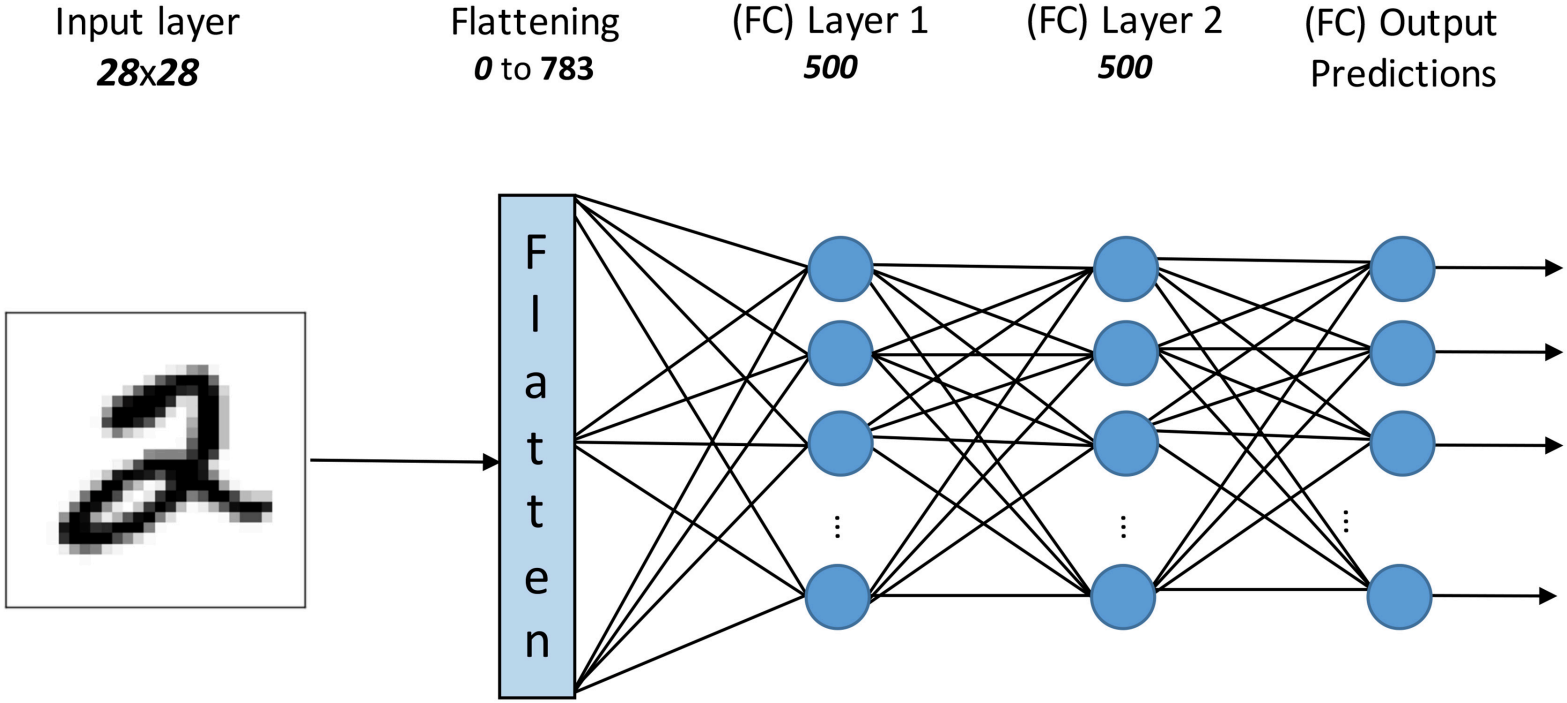
The topology of the fully connected network as trained by O'Connor et al. (2013).

To demonstrate that the proposed methodology can be used to fine-tune (optimize) the performance of an already trained SNN we utilized the fully connected network from O'Connor et al. (2013). This particular network consists of two hidden layers, each with 500 neurons as seen in Figure 4, and was trained on the MNIST dataset with the Contrastive Divergence (CD) algorithm (Hinton et al., 2006) using Siegert neurons (Siegert, 1951), which are a rate-based approximation of Integrate-and-fire neuron models.

This network has been reported to achieve a classification accuracy of 95.2% using the Siegert neurons (frame-based ANN) and 94.09% with LIF neurons (O'Connor et al., 2013). In addition, this SNN has been thoroughly investigated for its robustness to input noise and bit resolution requirements (Stromatias et al., 2015b) and has been implemented on various hardware platforms (Neil and Liu, 2014; Stromatias et al., 2015a) with success.

For this paper we are going to remove the fully connected output layer and replace it with the SNN classifier trained using the methodology described in the following sections.

### 2.4. Neuron model

**Figure 5:**
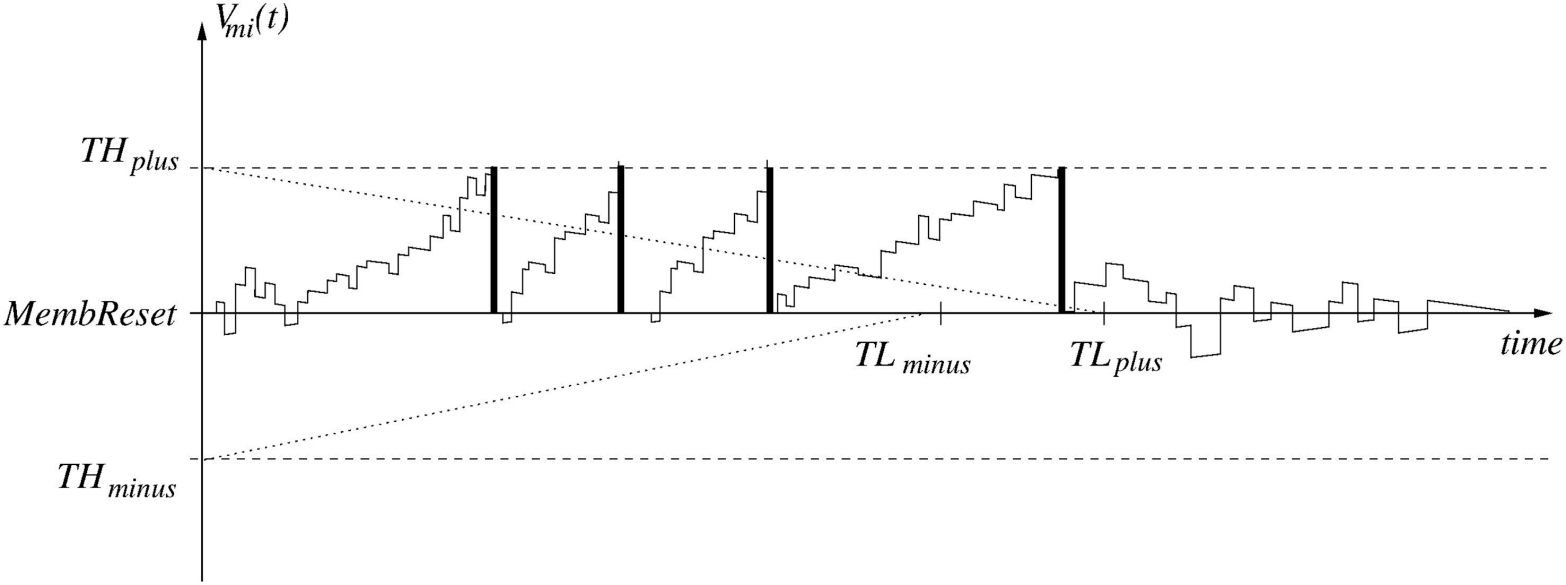
The evolution and spike generation sequence for a spiking neuron with linear leakage and refractory period.

The neuron model used is purely event-driven. Neurons' internal states, also called membrane voltages, are updated in response to an incoming event. The model uses linear leakage and delta-dirac (instantaneous) synapses. This neuron model is described in great detail by Pérez-Carrasco et al. (2013), while hardware implementations of this model have been reported by Camunas-Mesa et al. (2011, 2012) and Serrano-Gotarredona et al. (2015). A typical time evolution of a neuron membrane voltage *V*_*mi*_(*t*) is shown in Figure 5. All neurons in a layer share the same parameters *MembReset* (neuron resting level), *TH*_*plus*_ (positive threshold), *TH*_*minus*_ (negative threshold), and leakage rates. Leakage rates can be defined differently for *V*_*mi*_(*t*) > *MembReset* and *V*_*mi*_(*t*) < *MembReset*. When there is no input spike, membrane voltage *V*_*mi*_(*t*) is subject to leakage only,

(3) $$\begin{array}{l} \frac{dV_{mi}(t)}{dt}=-\frac{TH_{plus}-MembReset}{TL_{plus}},\;if\;V_{mi}(t)>MembReset\\ \frac{dV_{mi}(t)}{dt}=+\frac{MembReset-TH_{minus}}{TL_{minus}},\;if\;V_{mi}(t)<MembReset \end{array}$$

The event-driven simulator MegaSim only updates the leakage when a new input event is received. For this, each neuron stores the time of its latest input event within its state variables. After updating the leakage, the neuron state *V*_*mi*_(*t*) is updated instantaneously by adding/subtracting the synaptic weight of the connection of the incoming spike. When a neuron reaches its positive threshold *TH*_*plus*_, it generates a positive output spike and is reset to *MembReset*. If it reaches its negative threshold *TH*_*minus*_, it generates a negative output spike and is reset as well. This way, output spikes are “signed.” The sign of a presynaptic spike is combined with the sign of the synaptic weight to define the sign of the update at the post-synaptic neuron. Therefore, the present neuron model is a “signed” neuron model.

It is possible to degenerate this signed model into one that only generates positive spikes (or only negative spikes). This can be done, for example, by setting the negative threshold *TH*_*minus*_ to a very high number (like the limit of numerical precision in the simulator). In our experiments, the classifier (FC) always uses this degenerated positively-signed neuron model. This way it behaves like a LIF neuron with limited precision and linear leakage. For the convolution layer (C1) neurons, we will use symmetric thresholds (*TH*_*minus*_ = −*TH*_*plus*_) and leakage rates (*TL*_*plus*_ = *TL*_*minus*_) but negative spikes are disabled (*TH*_*minusInfo*_ = 0, see the Supplementary Material). Upon reaching the negative threshold (*TH*_*minus*_) the membrane resets to its resting state (*MembReset*), but no negative event is communicated. We found that this configuration produces slightly better results and keeps the neuron model identical to previously published work of software and hardware implementations (Camunas-Mesa et al., 2011, 2012; Pérez-Carrasco et al., 2013; Serrano-Gotarredona et al., 2015). In the Supplementary Materials we show the pseudo codes of the neuron models of the convolutional layer and of the classifier fully connected layer.

### 2.5. Methodology for training an event-driven classifier

For this work both SNN architectures have already extracted features. For the case of the ConvNet topology (Figure 2) this was done by using a “sufficiently rich” set of pre-programmed feature detection filters, as seen in Figure 3, for the C1 layer. For the case of the fully connected network (Figure 4), the 2 hidden layers were trained using the unsupervised methodology described in O'Connor et al. (2013). The only weights that are here subject to training are the weights of the fully-connected spiking classifier (output layer).

However, here training is not performed in the spiking domain. The method we used in this work consists of the following steps:

1.- Provide the stimulus spike sequence of each sample to the input of the SNN (layer 1), and build an analog vector representation (a “frame”) for each sample by building a normalized histogram of the counting events at the output of the “Flatten” module for the ConvNet topology and output of the second layer for the fully connected topology.
2.- Use these vectors (frames) to train a fully connected (non-spiking) classifier using Stochastic Gradient Descent (SGD).
3.- Use a scaled version of the learned classifier weights for the spiking classifier.

One parameter that in principle should affect both training and testing is the leakage rate. Depending on the leakage rate value, the normalized histograms may change slightly and therefore the learned weights as well. Consequently, testing should be done using the same leakage rate used for obtaining the normalized histograms. As a matter of fact, many works reported in the literature on Spiking Neural Networks do not use leakage (Diehl et al., 2015; Rueckauer et al., 2016) or use leakages in the order of seconds (O'Connor et al., 2013). However, if there is no leakage, after providing the spikes representing an input pattern, all neurons need to be reset to be ready for the next input. In this case, input samples have to be provided sample by sample (SBS) and the system needs to be reset between new sample presentations. In a real scenario setup (for example, a moving robot) the system does not know when a new sample comes in, and should work rather in a continuous mode. This is when leakage comes in, and neural activity fades away some time after input pattern activation. In this situation there is no need to reset neuron states and the system operates in a continuous manner. Consequently, if there is non-zero leakage we can present the input patterns one after the other, as long as there is enough inter-sample time to allow the neuron states to fade to their resting state. In this case, we can pass all symbols as a unique input spiking sequence. We call this scenario “One Pass” (OP). For the rest of the paper we will be distinguishing between SBS (zero leakage) and OP (non-zero leakage) setups, for both training (this is, obtaining the normalized histograms) and testing. We noticed that, in general, SBS performs slightly better than OP.

Next we describe in more detail each of the three steps followed for the training procedure.

#### 2.5.1. From events to frames

Using the spiking simulator MegaSim we collect the spike sequences at the output of module “Flatten” in the ConvNet topology and the second layer for the fully connected topology, in order to create a normalized spike histogram for each sample. For each of the neurons in layer “Flatten” (see Section 2.3), we count the total number of spikes. The resulting histogram is normalized with respect to the maximum value, resulting in an analog vector with N components within the interval [0,1], N is the number of outputs for the flatten layer. These resulting spike histograms vary depending on the data set, the parameters of layer C1, and if there is leakage or not.

#### 2.5.2. Training

The normalized histograms of the spike-counts of the Flatten layer (Figure 2) for each input sample are converted to an analog vector (frame). This is repeated for both the training and testing set in order to create a new data set that will be used to train the classifier.

The classifier used for this work is *Softmax* regression trained with mini-batch stochastic gradient descent (MSGD) (Bottou, 2010) without biases. Outputs of the classifier **Y** = (*Y*_1_, ⋯*Y*_*k*_, ⋯ ) represent the scores or probabilities for each class *K*. The *Softmax* activation function results in the following class *K* output prediction probability for a given input vector **x**_*i*_

(4) $$\begin{array}{r} Y_{k}(x_{i},W)=\frac{e^{W_{k}x_{i}}}{\sum_{j}e^{W_{j}x_{i}}} \end{array}$$

where *W* is the weight matrix, and **x**_*i*_ is the input vector fed to the classifier provided by layer Flatten. Vector **W**_*k*_ is the weight matrix line representing class *K*. Note that the probabilities of all classes add to one.

The cost function to be minimized by the MSGD algorithm is the Negative Log-Likelihood Loss (NLL), which is described as:

(5) $$\begin{array}{r} C=-\frac{1}{D}\overset{\left|D\right|}{\underset{i}{\sum }}\overset{\left|K\right|}{\underset{j}{\sum }}I_{i}\left(j\right)\log\left(Y_{j}\left(X_{i},W\right)\right) \end{array}$$

where *D* is the mini-batch, *K* is the set of classes, *I*_*i*_(*j*) = 1 for *j* = *L*_*i*_ and 0 otherwise. *L*_*i*_ is the target output (label) for sample *i*, and *Y*_*j*_ is the output of the Softmax unit *j*.

The purpose of the SGD is to minimize the cost function *NLL* by updating the parameters of the model *W* ∈ ℜ^*h*×|*K*|^, where *h* is the size of the Flatten layer and K is the number of classes. The MSGD updates the weight matrix after each mini-batch by computing the gradient with respect to the weights of the objective function,

(6) $$W=W-\eta \nabla_{W}C$$

where η is the learning rate, which determines the size of the update steps taken to reach a minimum. For this work we used a fixed learning rate and fixed number of epochs.

The predicted class *y*_*i*_ of the model for a given input **x**_*i*_ is taken as the unit that has the maximum activation and is described by

(7) $$\begin{array}{r} y_{i}=argmax_{k}\left\{Y_{k}\left({\text{x}}_{i},W\right)\right\} \end{array}$$

#### 2.5.3. From frames back to events

After the training process is over the learned weights are scaled by a constant integer *K*. This integer should be of the order of the threshold of the fully connected classifier spiking neurons *TH*_*FC*_. As we will see in Section 3, we have set *TH*_*FC*_ = *K*, with *K* always equal to 10,000,000.

## 3. Results

We performed extensive training and testing on the different data sets described in Section 2.1, in order to characterize the effectiveness of the spiking classifier. We measure the effectiveness of the spiking classifier by what we call here “**Classifier Loss**.” We define “**Classifier Loss**” as the difference in recognition performance between the accuracy obtained with the ANN frame-based classifier and that obtained after mapping the classifier to its SNN version. If “**Classifier Loss**” is negative, there is a degradation when going from ANN to SNN, and if it is positive there is an improvement.

Table 2 shows the different parameters used for the different trials. Since the focus of this work is on studying and characterizing the classifier, we tried to keep the first convolution layer as similar as possible among all data sets. However, depending on the nature of data set we had to adjust a few parameters and options specifically for each. The number of Feature Maps (FM), or C1 convolutions, was always kept at 18. The feature map size, however, was changed between 28 × 28 and 128 × 128 to adapt to the input space of each data set. Kernel size also varies from 7 × 7 to 21 × 21. Some meta-parameters (like selected time slices for building histograms) were adjusted manually at the very beginning by quickly playing with some training set samples. We never used any test set result to fine tune any parameter, because this would “leak” information about the test set to the system (Nowotny, 2014). All meta-parameters were decided from the beginning and then never touched again. This was because our goal was not to optimize the overall system accuracy, but the “**Classifier Loss**.”

**Table 2:** Parameters used for the C1 Convolution Layers and the FC Classifiers.

**Data set**	**Input**	**C1 Convolutions**							**FC Classifier**		
	**Input size**	**# of FMs**	**FM size**	**Kernel size**	***TH*_*plus*_**	***TH*_*minus*_**	***TL*_*plus*_ (OP)**	***TL*_*minus*_ (OP)**	**# of neurons**	***TH*_*plus*_**	***TL*_*plus*_ (OP)**
Synthetic MNISTs	28 × 28	18	22 × 22	7 × 7	1.2 × 10^8^	−1.2 × 10^8^	1.2 × 10^4^	1.2 × 10^4^	10	10^7^	1.2 × 10^4^
N-MNIST	34 × 34	18	28 × 28	7 × 7	2 × 10^7^	−2^31^	10^6^	10^6^	10	10^7^	10^6^
MNIST-DVS	128 × 128	18	108 × 108	21 × 21	8 × 10^8^	−2^31^	1.5 × 10^4^	−1.5 × 10^4^	10	10^7^	1.2 × 10^4^
Slow-Poker-DVS	128 × 128	18	114 × 114	15 × 15	1.5 × 10^7^	−1.5 × 10^7^	1.5 × 10^4^	1.5 × 10^4^	4	10^7^	1.5 × 10^5^
Fast-Poker-DVS	32 × 32	18	26 × 26	7 × 7	1.5 × 10^7^	−2^31^	1.5 × 10^4^	1.5 × 10^4^	4	10^7^	1.5 × 10^5^

The positive and negative thresholds of the C1 layer neurons were set symmetrically and for the classifier FC we set a maximum negative threshold to make it behave like a population of LIF neurons, as discussed in Section 2.4. We observed that for both synthetic MNIST data sets (with latency or Poisson encoding), and for the DVS data sets, we obtained slightly better results when using this configuration for neurons in layer C1. For the FC Classifier we always used unsigned neurons with threshold at 10M. The leakage parameters *TL*_*plus*_ and *TL*_*minus*_ are only used when data is presented in OP (One Pass). When data is presented SBS (Sample by Sample) all leakages were set to “0.”

The performance results obtained are summarized in Table 3. The first column is the “Training Slice Size” (TSS). This refers to the time slices into which the data set sample sequences were cut to present them to layer C1 and build the normalized histograms at layer Flatten for training in the frame domain. For all MNIST types of data sets we always used the full sample spiking sequence. This duration was equal to 255 μs for both synthetic MNISTs (Latency and Poisson), between 2 and 3 s for MNIST-DVS and about 300 ms for N-MNIST. For the Poker-DVS data sets we did not use the full sample sequence. For the Slow-Poker-DVS, as mentioned in Section 2, all sample recordings were split into 100 ms slices, while for the Fast-Poker-DVS, we tried three different slice sizes (2, 5, and 10 ms), because the performance was very sensitive to the slice size.

**Table 3:** Summary of performance results.

**Data set**	**TSS**	**Train and Test SBS**						**Train OP**				
		**ANN (frames)**	**SNN**	**Classifier Loss**	**Average input sample activity**	**Average total sample activity**	**Latency (μs)**	**ANN (frames)**	**SNN Test OP**	**Classifier Loss**	**SNN Test SBS**	**Classifier Loss**
Latency	Full	98.45	98.42	−0.03	151.12	957.37	10.89	98.41	98.39	−0.02	98.37	−0.04
Poisson	Full	98.15	98.20	−0.05	1,000	16,070.28	11.38	98.3	98.25	−0.05	98.32	−0.02
N-MNIST	Full	97.77	97.23	−0.54	4,203.61	29,9425.57	2,901.10	97.76	97.08	−0.68	97.09	−0.67
MNIST-DVS	Full	97.3	97.25	−0.05	73,520.96	139,707.66	58,918.25	97.95	97.9	−0.05	97.95	0.00
Slow-Poker-DVS	100 ms	99.77	99.70	−0.07	1,418.94	154,483.18	774.27	98.95	98.59	−0.36	98.88	−0.07
	2 ms	94.44	100	+5.66		231662.29	100.67	92.78	100	−7.22	100	+7.22
Fast-Poker-DVS	5 ms	95.00	94.65	−0.35	2539.77	224598.88	124	95	93.13	−1.87	93.89	−1.11
	10 ms	100	83.97	−6.03		222681.92	126.21	100	85.50	−14.5	85.50	−14.5

The results in Table 3 are separated into two groups, the first for training and testing without any leakage with inputs in SBS format, and the second trained with leakage with inputs in OP format. In this second case, we show tests with inputs in both OP and SBS formats. Columns “ANN (frames)” refers to the accuracy obtained in the frame domain when training the classifier with the normalized histograms as inputs. These numbers will be used as the baseline reference for comparing the performance of the classifier spiking version. Columns “SNN” show the accuracy obtained when mapping the learned weights to the spiking network, and columns “**Classifier Loss**” indicate our performance figure defined above. For the cases of training and testing without leakage (SBS) we also provide the average number of events per input sample (Average Input sample Activity), the average total number of per samples produced within the full network including the input (Average Total sample Activity), and the “latency” defined as the time difference between the first spike of the input layer and the first output spike from the Classifier. The training parameters for example the number of epochs and the learning rate (η) were kept fixed to 1,500 epochs and 0.1, respectively, for every data set.

**Figure 6:**
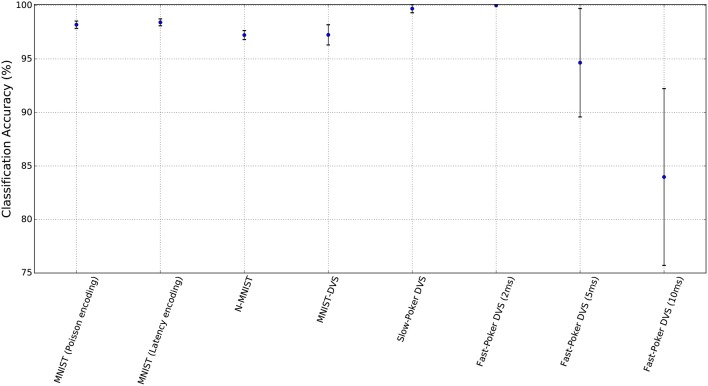
Classification accuracies of the SNNs for each data set together with their corresponding confidence intervals.

Figure 6 summarizes the classification accuracies of the test set for the different data sets and their confidence intervals. The confidence intervals were calculated for a confidence level of 0.99 and assuming that the test samples are statistically independent. These confidence intervals give an idea of the expected accuracy depending on the method (Isaksson et al., 2008; Nowotny, 2014). It can be seen that the confidence intervals are closely related to the test set size and the number of successes.

**Figure 7:**
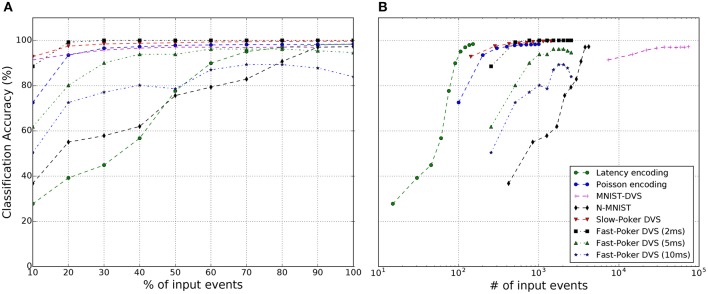
The classification accuracy of the SNNs for each data set as **(A)** a function of average percentage of input events per symbol presentation, and **(B)** average absolute number of input events per symbol presentation.

In the following sub-sections we indicate more specific details for each data-set. Figure 7 reveals for each data-set how recognition accuracy evolves during one symbol presentation (on average) as function of the running number of input events. This is, in an event-driven system, its internal states and its outputs evolve as input events are provided, event by event. Therefore, some output can be available during symbol presentation and it may change with each new input event during presentation of the input event sequence. With few input events, output accuracy is low. But as more input events arrive for a given input symbol, the recognition accuracy keeps improving, until typically reaching a plateau. Since the data-sets we are using have a quite diverse average number of events per symbol, we show in Figure 7A the recognition accuracy evolution with the percentage of input events per symbol, while Figure 7B shows it as a function of the absolute number of input events (in logarithmic scale).

### 3.1. Synthetic MNISTs

For the synthetic MNIST spiking data sets (Latency and Poisson) we used the full original frame-based handwritten digit data set consisting of 70,000 samples out of which 60,000 are used for training and 10,000 testing. The training batch size was kept fixed to 500 samples. In order to have a fair comparison between the Poisson encoding and the Latency encoding, the stimulus duration for both was set to 255 μs. The threshold of the C1 neurons was manually and coarsely adjusted to maximize classification accuracy. The same parameters were used for the FC Classifier layer for both synthetic encodings as can be seen in Table 2. For the synthetic MNIST data sets we observed typically a **Classifier-Loss** between −0.02 and −0.05%, except for the Poisson case if training with leakage but testing without leakage. In this case we observed a slight improvement of +0.02%. Moreover, a comparison between the Latency and Poisson encoding, revealed that the Latency encoding does indeed generate less events, as expected. However, as seen in Figures 7A,B the Poisson encoding requires less than 50% of the total spikes to reach close to the maximum score, whereas Latency encoding requires 100% of the input spikes to achieve the same effect. This makes sense since in the Latency encoding there is only one spike per pixel, while in Poisson encoding a pixel fires multiple times.

### 3.2. N-MNIST

The N-MNIST data set comprises 70,000 samples, 60,000 for training and 10,000 for testing. Each sample is composed of 3 sequential saccades of about 100 ms each. Therefore the whole symbol duration is of about 300 ms. The training batch size was set to 500 samples. The training and testing neural network parameters can be seen in Table 2. For the SBS result there is a loss in the accuracy of −0.54%. when training OP we observed a slightly higher loss in the accuracy, −0.68% testing OP and −0.67% testing SBS. Therefore the best SNN accuracy result obtained is 97.23% when performing the training and testing SBS.

### 3.3. MNIST-DVS

For the MNIST-DVS data set, we use the full DVS 128 × 128 pixel resolution. The data set comprises 10,000 MNIST digit samples, out of which 8,000 are used for training and 2,000 for testing. The training batch size was reduced to 20 samples. The threshold of the C1 neurons was manually adjusted to improve classification accuracy (see Table 2). In this case the C1 convolutional kernel size selected was 21 × 21. For this data set we observed a loss in the accuracy of 0.05% for the SBS experiments and for training and testing OP. There is no accuracy loss training OP and testing SBS. The best SNN accuracy result is 97.95%. There are no accuracy results reported in literature for this data set, however, it is interesting to note that using a smaller data set than the N-MNIST, 10,000 samples compared to 70,000 samples of the N-MNIST, we achieve a slightly better accuracy result.

### 3.4. Slow-poker-DVS

Data set uses the full DVS resolution. There is a total of 6,751 samples, 5,402 samples are used for training and 1,349 for testing. The training batch size was 50 samples and the convolutional kernel size was 15 × 15 (see Table 2). Each sample duration was set to 100 ms. The best SNN accuracy performance obtained was 99.70, 0.07% lower that the classifier result when performing the training and testing SBS. The loss is higher when training and testing OP, -0.36% and the accuracy of the classifier also dropped to 98.95%.

### 3.5. Fast-poker-DVS

**Figure 8:**
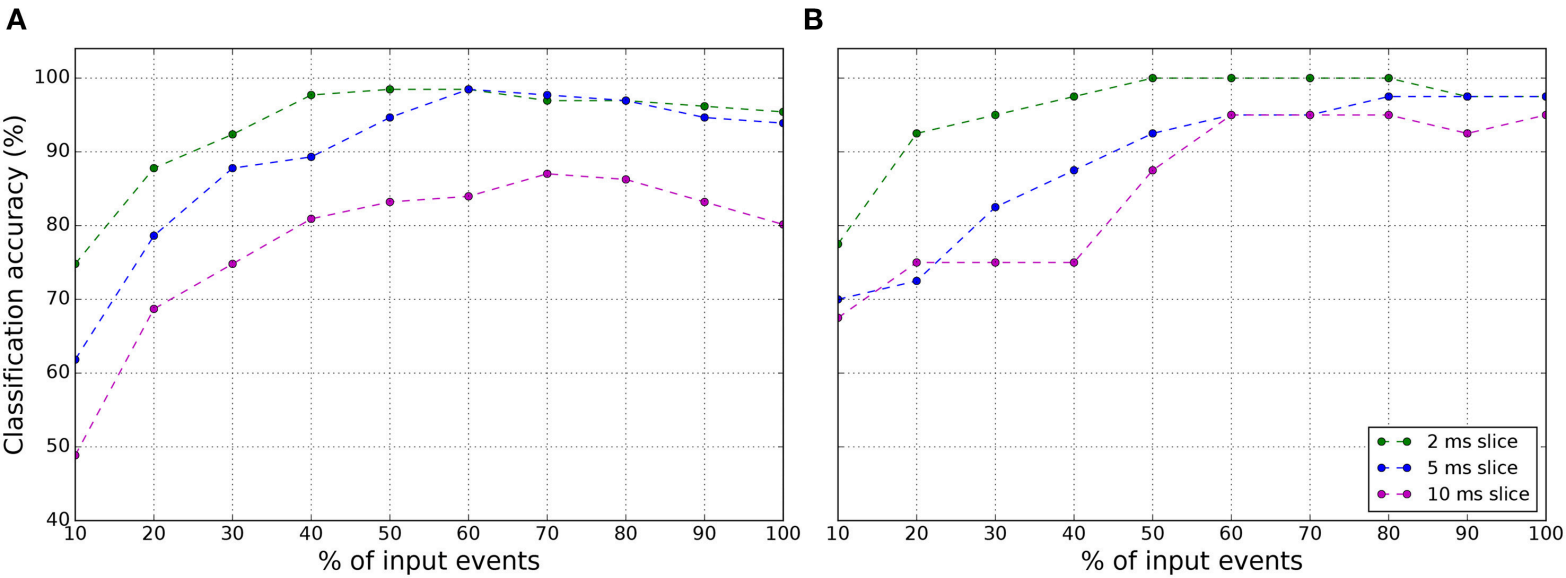
Classification accuracy of the SNN using **(A)** the LOOCV method over the 131 samples Fast-Poker-DVS data set and **(B)** the LOOCV method over the 40 samples Fast-Poker-DVS data set.

The number of samples for this data set is limited to 131 samples. Each sample duration is ~10–30 ms. The approach followed with this data set to increase the number of samples and improve the accuracy results was to divide the sample representation in different time slices for training the classifier using Softmax regression. For example, using time slices of 2 ms, the training set for the classifier extends to 776 samples and 192 for testing. Even though the classifier training was done using these time slices, the SNN testing was always done over the whole 131 sample representation, the 10–30 ms. Therefore a 100% of accuracy corresponds to 131 samples correctly identified. The results in Table 3 shows that increasing the time slice increases the classifier accuracy, but decreases the SNN accuracy. The classifier accuracy increases because with larger time slices, the training and testing samples are more similar, this is not happening with smaller time slices. However, for SNN testing, finding a longer time slice pattern in the whole test sample is more difficult and therefore it behaves better with smaller time slices as 2 ms. It is worth noting that by using a 2 ms time slice we achieve a 100% SNN accuracy for both training SBS and OP. However, to be more correct, these results correspond to training error, because the training slices were part of the whole sample of the testing set. To obtain a more correct recognition accuracy, the leave-one-out cross-validation (LOOCV) method was applied to this data set obtaining the recognition accuracy shown in Figure 8A. The recognition accuracy reaches its top when the 50–60% of the events of the samples are displayed. For both, 2 and 5 ms slice, the best recognition accuracy is 98.47 at 60% of the events. This experiment was also done with the Fast-Poker-DVS of 40 samples data base for the aim of comparing them with the results in Orchard et al. (2015b) and Lagorce et al. (2016). Orchard et al. (2015b) and Lagorce et al. (2016) obtained 97.5 and 100% recognition success, respectively. In this work, for the 40 cards, applying the LOOCV method and using 2 ms slices the classification accuracy reaches 100% when presented 50–80% of the inputs events, as seen in Figure 8B.

### 3.6. SNN fine-tuning

The SNN shown in Figure 4 was trained assuming Poisson distribution for the input stimuli and was originally presented in O'Connor et al. (2013) achieving a score of 95.2% in the frame domain and 94.09% with a software SNN simulator. Since then this SNN has been successfully implemented in various platforms (Neil and Liu, 2014; Stromatias et al., 2015a,b), with the highest score 95% reported in Stromatias et al. (2015a). Here, we removed the output layer and trained a new classifier by taking advantage of the feature extraction done by the previous two layers. Table 4 summarizes the results. The new frame-based classifier achieves a 97.24%, while the SNN accuracy increased from 95.26 to 97.25%. The difference between the frame-based classifier and the SNN is in the order of 0.01%.

**Table 4:** Summary of fine-tuning an already trained SNN.

	**Classifier (frames) (%)**	**SNN (MegaSim) (%)**
Original Netowrk	95.2 (O'Connor et al., 2013)	95.26
Fine-tuned (this work)	97.24	97.25

### 3.7. Summary of results and comparison with related work

**Figure 9:**
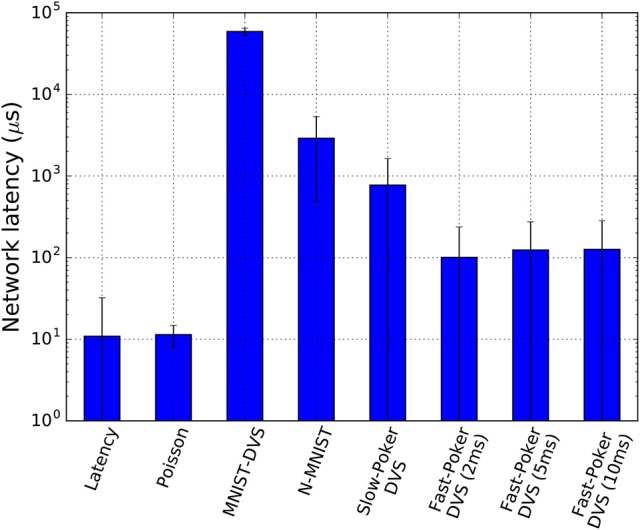
The mean and standard deviation of the classification latency of the SNNs for each data set.

Table 3 and Figure 7 summarize the results of this work for all data sets. More specifically, Figure 7A shows how the classification accuracy of an SNN improves as a function of the percentage of input events. This seems to be consistent with all data sets, both synthetically generated (Latency and Poisson encoding) and from a DVS sensor, and is in accordance with previously published studies (Neil and Liu, 2014; Diehl et al., 2015; Stromatias et al., 2015b). With Fast-Poker-DVS data set, there is a decrease in the performance in the last 20% of the input events due to the deformation of the card symbol when it disappears. Figure 7B presents the classification accuracy as a function of the absolute number of input events, in log scale, for the different data sets. This information is useful because in neuromorphic systems the overall energy consumption depends on the total number of events that are going to be processed (Stromatias et al., 2013; Merolla et al., 2014; Neil and Liu, 2014) and this might have an impact on deciding which data set to use based on the energy and latency constraints Figure 9, presents the network latency for each data set. We identify network latency as the time lapsed from the first input spike to the first output spike.

Table 5 presents a comparison of the current work with results in the literature on the MNIST data set and SNNs. The current state-of-the-art results come from a spiking CNN with 7 layers and max-pooling achieving a score of 99.44% and from a 4 layer spiking FC network achieving a score of 98.64% (Diehl et al., 2015). Both approaches were trained offline using frames and backpropagation and then mapped the network parameters to an SNN. However, even though this approach works very well with Poisson spike-trains or direct use of pixels, performance drops significantly with real DVS data. In addition, a direct comparison is not fair because the focus of this paper was to develop a classifier that works with both synthetic and DVS data and not to train a complete neural network with multiple layers.

**Table 5:** Comparison of classification accuracies (CA) of SNNs on the MNIST data set.

**Architecture**	**Neural coding**	**Learning-type**	**Learning-rule**	**CA (%)**
Spiking RBM (Neftci et al., 2015)	Poisson	Unsupervised	Event-Based CD	91.9
FC (2 layer network) (Querlioz et al., 2013)	Poisson	Unsupervised	STDP	93.5
FC (4 layer network) (O'Connor et al., 2013)	Poisson	Unsupervised	CD	94.1
FC (2 layer network) (Diehl and Cook, 2015)	Poisson	Unsupervised	STDP	95.0
Synaptic Sampling Machine (3 layer network) (Neftci et al., 2016)	Poisson	Unsupervised	Event-Based CD	95.6
FC (4 layer network) (this work(O'Connor et al., 2013))	Poisson	Supervised	Stochastic GD	97.25
FC (4 layer network) (O'Connor and Welling, 2016)	–	Supervised	Fractional SGD	97.8
FC (4 layer network) (Hunsberger and Eliasmith, 2015)	Not reported	Supervised	Backprop soft LIF neurons	98.37
FC (4 layer network) (Diehl et al., 2015)	Poisson	Supervised	Stochastic GD	98.64
CNN (Kheradpisheh et al., 2016)	Latency	Unsupervised	STDP	98.4
CNN (Diehl et al., 2015)	Poisson	Supervised	Stochastic GD	99.14
Sparsely Connected Network (×64) (Esser et al., 2015)	Poisson	Supervised	Backprop	99.42
CNN (Rueckauer et al., 2016)	Poisson	Supervised	Stochastic GD	99.44
CNN (this work)	Latency	Supervised	Stochastic GD	98.42
CNN (this work)	Poisson	Supervised	Stochastic GD	98.20

Table 6 gathers the results in literature using the N-MNIST data set and SNN. The best classification accuracy reported is 98.66% using a FC 3 layer network (Lee et al., 2016). Using a CNN, this work reports the best classification accuracy 97.77% until now. Again, the focus of this paper is not beating the classification accuracy, there is no optimization done to improve the performance, but to provide a valid SNN classifier training method with an insignificant **Classifier Loss** compared to frame based classification accuracy.

**Table 6:** Comparison of classification accuracies (CA) of SNNs on the N-MNIST data set.

**Architecture**	**Preprocessing**	**Learning-type**	**Learning-rule**	**CA (%)**
CNN (Orchard et al., 2015b)	None	Unsupervised	HFirst	71.15
FC (2 layer network) (Cohen et al., 2016)	None	Supervised	OPIUM (van Schaik and Tapson, 2015)	92.87
CNN (Neil and Liu, 2016)	Centering	Supervised	–	95.72
FC (3 layer network) (Lee et al., 2016)	None	Supervised	Backpropagation	98.66
CNN (this work)	None	Supervised	SGD	97.77

Finally Table 7 shows the literature results for the 40 card fast-poker-dvs data set. With this work, we demonstrate that 100% of classification accuracy is obtained using LOOCV method.

**Table 7:** Comparison of classification accuracies (CA) of SNNs on the 40 cards Fast-Poker-DVS data set.

**Architecture**	**Learning-type**	**Learning-rule**	**CA (%)**
CNN (Pérez-Carrasco et al., 2013)	Supervised	Backprop	90.1 − 91.6
CNN (Orchard et al., 2015b)	Unsupervised	HFirst	97.5 ± 3.5
CNN (Lagorce et al., 2016)	Supervised	HOTS	100
CNN (this work)	Supervised	Stochastic GD	100

## 4. Discussion

In this paper we have presented a novel method for training a classifier and converting it to an SNN. One of the main advantages of the proposed technique is that it is able to work with both synthetically generated data, used frequently in previous studies (Masquelier and Thorpe, 2007; O'Connor et al., 2013; Neftci et al., 2014; Diehl and Cook, 2015; Diehl et al., 2015; Kheradpisheh et al., 2016), as well as with real DVS data from a neuromorphic vision sensor (Lichtsteiner et al., 2008; Posch et al., 2011; Serrano-Gotarredona and Linares-Barranco, 2013). Recent works reported very good results while using synthetically generated events, however, this performance does not hold true when switching to DVS data, as a large drop in the classification accuracy is observed.

Here we used a single convolutional layer with the same number of feature maps and the same 18 hand-coded Gabor-like kernels, in order to prove that our methodology works with both synthetic and DVS data. We did not try to optimize the kernels for each data set as this would be out of the scope of this work, only the dimensions of the feature maps changed according to the size of each data set. The importance of the results is to show the small difference between the frame-based classifier accuracy result and the SNN classifier accuracy results. We also considered cases where leakage in neurons is activated, as this is more suitable for real-world applications.

We have shown that for synthetic data there is a loss of 0.03% in the classification accuracy when compared to the frame-based classifier, while for the N-MNIST data set the higher loss between the frame-based classifier and the SNN is in the order of 0.68%. On the N-MNIST the SNN achieved a score of 97.77%, which at the time of writing is the highest score for a convolutional SNN using the N-MNIST raw data. We also tested the classifier with the Fast-Poker-DVS data set, 40 cards, and obtained 100% of accuracy by separating the data set into training and testing sets. Furthermore, we extend this data set to a slightly bigger data set, 131 cards, showing a classification accuracy of 98.47%.

In addition, we presented a comparison between the different encodings and data sets in terms of classification latency and total number of events. This comparison is important because for neuromorphic platforms the energy consumption depends on the events to be processed (Stromatias et al., 2013; Merolla et al., 2014), and depending on the application one encoding might be more preferable than the other. As an example, mobile and robotic platforms that have limited energy resources and require fast responses might benefit from using Intensity-to-Latency encoding, since it produces the lowest network activity and output latency compared to all the data sets presented in this work.

The proposed methodology has also been used to fine-tune an already trained SNN. We showed that by using the features learned in an unsupervised manner using the CD algorithm (O'Connor et al., 2013) and only retraining the classifier, the classification accuracy improved by 2%. This result suggests that our classifier can be used in cases where features are extracted in an unsupervised manner, either based on biologically-plausible plasticity rules such as STDP (Bichler et al., 2012; Roclin et al., 2013; Diehl and Cook, 2015; Kheradpisheh et al., 2016) or event-based implementations of CD (Neftci et al., 2014, 2016).

## Author contributions

ES and MS conceived the idea of the proposed classifier. ES designed, and conducted the experiments with the synthetic data and analyzed the results. MS designed, performed the experiments with the DVS data sets and analyzed the results. ES, MS, and BL wrote the paper. BL wrote the MegaSim simulator code, and ES contributed new codes and python layers for systematic runs of repeated simulations and data analyses. BL contributed to the conception and design of the experiments, data analysis, and presentation of the work. TS provided an improved DVS sensor with which the most recent recordings were made.

### Conflict of interest statement

The authors declare that the research was conducted in the absence of any commercial or financial relationships that could be construed as a potential conflict of interest.

## Footnotes

^1^A synaptic event is defined as a spike hitting a single synapse.

^2^On the other hand, training the full network in the ANN domain and mapping it to SNN is a well known method.

^3^https://bitbucket.org/bernabelinares/megasim

## Supplementary material

Supplementary material is included showing the pseudo-codes of the neuron models used in the algorithms.
