# Supplementary material for: An Event-Driven Classifier for Spiking Neural Networks Fed with Synthetic or Dynamic Vision Sensor Data
Source: Front Neurosci. 2017 Jun 28;11:350. doi: 10.3389/fnins.2017.00350 (PMC5487436; doi:10.3389/fnins.2017.00350)
Supplement: Supplementary file 1 [file DataSheet1.pdf]

frontiers

Supplementary Material: An Event-Driven Classifier for Spiking Neural Networks fed with Synthetic or Dynamic Vision Sensor Data

Evangelos Stromatias [†,1], **Miguel Soto** [†,1], **Teresa Serrano-Gotarredona**[1], **and Bernabé Linares-Barranco** [*,1]

*Correspondence:
Bernabé Linares-Barranco
bernabe@imse-cnm.csic.es

1 SUPPLEMENTARY DATA

Details of the algorithms for the neuron models are given here. Algorithm 1 presents the convolutional module, while Algorithm 2 shows the fully-connected module as implemented in MegaSim.

2 CALCULATING THE CONFIDENCE INTERVALS

The confidence intervals of the mean test error are computed using the following formula:

$$\hat{\mu} \pm z^{*}(\sqrt{\frac{\hat{\mu}(1-\hat{\mu})}{N}}) \tag{S1}$$

where $\hat{\mu}$ is the estimated test error, N is the test set size, and z^{*} depends on the level of confidence. For this work we used a 99% confidence interval so z^{*} is set to 2.578.

function module_conv $(event_{in}, t_{actual})$;
Input : AER input event as an integer array of 3 elements: $event_{in}$, and t_{actual} time of the input AER event

```
THplus, THminus,                          ▷ Positive/negative threshold
TLplus, TLminus,                          ▷ Positive/negative leakage
THPlusInfo, THMinusInfo,                  ▷ Enable events
MembReset,                                ▷ Membrane reset
T_refract,                                ▷ Refractory period
FmYSize, FmXSize,                         ▷ Feature map size
kYSize, kXSize,                           ▷ Kernel size
DX, DY                                    ▷ Kernel disposition
← get_fm_params();
for y = event_in[1] + DY, y < min(FmYSize, event_in[1] + DY + kYSize, y++ do
    for x = event_in[0] + DX, x < min(FmXSize, event_in[0] + DX + kXSize), x++ do
        Vm_i ← get_neuron_membrane(x, y);
        t_i_lastoutspike ← get_neuron_last_out_spike(x, y);
        t_i_lastinspike ← get_neuron_last_in_spike(x, y);
        if t_i_lastoutspike + T_refract ≤ t_actual then
            if Vm_i ≥ MembReset then
                if TLplus ≠ 0 then
                    Vm_i ← max(MembReset, Vm_i − ((THplus − MembReset) ×
                      (t_actual − t_i_lastinspike) ) / TLplus);
                end
            else
                if TLminus ≠ 0 then
                    Vm_i ← min(MembReset, Vm_i − ((THminus − MembReset) ×
                      (t_actual − t_i_lastinspike) ) / TLminus);
                end
            end
            w ← get_weight(x, y);
            Vm_i ← Vm_i + w ;
            set_neuron_membrane(x, y, Vm_i);
            // Threshold checking
            if Vm_i ≥ THplus then
                if THplusInfo=1 then
                    GenerateSpike(x, y, +1);
                end
            end
            else if Vm_i ≤ THminus then
                if THminusInfo=1 then
                    GenerateSpike(x, y, −1);
                end
            end
        end
    end
end
```

Algorithm 1: The convolutional module in MegaSim

function module_fully_connected $(event_{in}, t_{actual})$;
Input : AER input event as an integer array of 3 elements: $event_{in}$, and t_{actual} time of the input AER event

```
THplus,                                                    ▷ Membrane threshold
TLplus,                                                    ▷ Membrane leakage
THPlusInfo,                                                ▷ Enable output events
MembReset,                                                 ▷ Membrane reset
T_refract,                                                 ▷ Refractory period
populationsize,                                            ▷ Population size
← get_population_params();
for n = 0, n < populationsize, n++ do
    Vm_i ← get_neuron_membrane(n,0);
    t_i_lastoutspike ← get_neuron_last_out_spike(n, 0);
    t_i_lastinspike ← get_neuron_last_in_spike(n, 0);
    if t_i_lastoutspike + T_refract ≤ t_actual then
        if Vm_i ≥ MembReset then
            if TLplus ≠ 0 then
                Vm_i ← max(MembReset, Vm_i − ((THplus − MembReset) ×
                  (t_actual − t_i_lastinspike) ) / TLplus);
            end
        else
            if TLplus ≠ 0 then
                Vm_i ← min(MembReset, Vm_i − ((INT_MIN − MembReset) ×
                  (t_actual − t_i_lastinspike) ) / TLplus);
            end
        end
        w ← get_weight(n, 0);
        Vm_i ← Vm_i + w ;
        set_neuron_membrane(n, 0, Vm_i);
        // Threshold checking
        if Vm_i ≥ THplus then
            if THplusInfo=1 then
                GenerateSpike(n, 0, +1);
            end
        end
    end
end
```

Algorithm 2: The fully-connected module in MegaSim
